# Serum biomarker analysis of collagen disease patients with acute-onset diffuse interstitial lung disease

**DOI:** 10.1186/1471-2172-14-9

**Published:** 2013-02-14

**Authors:** Shomi Oka, Hiroshi Furukawa, Kota Shimada, Hiromi Hayakawa, Naoshi Fukui, Naoyuki Tsuchiya, Shigeto Tohma

**Affiliations:** 1 Clinical Research Center for Allergy and Rheumatology, Sagamihara Hospital, National Hospital Organization, 18-1 Sakuradai, Minami-ku, Sagamihara, Kanagawa, 252-0392, Japan; 2 Department of Rheumatology, Sagamihara Hospital, National Hospital Organization, 18-1 Sakuradai, Minami-ku, Sagamihara, 252-0392, Japan; 3 Tokyo Metropolitan Tama Medical Center, 2-8-29 Musashi-dai, Fuchu, 183-8524, Japan; 4 Molecular and Genetic Epidemiology Laboratory, Faculty of Medicine, University of Tsukuba, 1-1-1 Tennodai, Tsukuba, 305-8575, Japan

**Keywords:** Collagen disease, Biomarker, Cytokine, Interstitial lung disease

## Abstract

**Background:**

Interstitial lung disease (ILD) is frequently associated with collagen diseases. The prognosis of acute-onset diffuse ILD (AoDILD) occurring in collagen disease patients is very poor. Here, we investigated serum biomarker profiles of AoDILD to find markers predicting outcome in patients with collagen diseases.

**Methods:**

A solid-phase antibody array was used for screening 274 biomarkers in pooled sera from collagen disease patients in the AoDILD state and in the stable state. Biomarkers in individual sera were detected without pooling by bead-based immunoassay.

**Results:**

The serum levels of matrix metalloproteinase (MMP)-1, tissue inhibitor of metalloproteinase (TIMP)-1, osteopontin, interleukin (IL)-2 receptor α (IL-2Rα), and IL-1 receptor antagonist were significantly increased in AoDILD, but TIMP-2, MMP-3, and eotaxin 2 levels were decreased. The MMP-3 to MMP-1 ratio was reduced in AoDILD state. This tendency was also observed in RA patients with AoDILD. Moreover, serum IL-6 level was significantly increased in the AoDILD state in patients with acute exacerbation of ILD (AE-ILD). Serum TIMP-1 and IL-2Rα levels were significantly increased in the AoDILD state in patients with drug-induced ILD (DI-ILD), whereas TIMP-2, MMP-3, and eotaxin 2 levels were decreased. The MMP-3 to MMP-1 ratio was reduced in AoDILD state in patients with DI-ILD. The serum TIMP-3, MMP-9, osteopontin, IL-2Rα, MMP-1, and MMP-8 levels were significantly increased in the AoDILD state in patients who subsequently died, whereas TIMP-2 and MMP-3 levels were decreased in those who survived. The MMP-3 to MMP-1 ratio was reduced in AoDILD state in patients who died, but not in those who survived.

**Conclusions:**

Serum biomarker profiles could represent prognosis markers for AoDILD in collagen diseases.

## Background

Interstitial lung disease (ILD) is characterized by interstitial inflammation of the lung and is frequently associated with collagen diseases, when it is designated collagen vascular disease-associated ILD (CVD-ILD). CVD-ILD is one of the major manifestations of collagen disease that influence the prognosis [1,2]. Acute-onset diffuse ILD (AoDILD) occurs in patients with collagen disease with or without underlying CVD-ILD [3]. AoDILD includes acute exacerbation of ILD (AE-ILD), drug-induced ILD (DI-ILD), and *Pneumocystis* pneumonia. The prognosis of AoDILD is quite poor. AE-ILD is due to collagen diseases *per se* and characterized by pathological findings of diffuse alveolar damage overlapping with chronic fibrotic lung. DI-ILD occurs frequently in rheumatoid arthritis (RA) patients treated with methotrexate. *Pneumocystis* pneumonia is defined by the presence of *P. jirovecii* organisms in the respiratory tract. However, the classification of AoDILD is not established.

The roles of several cytokines, chemokines, matrix metalloproteinases (MMPs) and tissue inhibitors of metalloproteinases (TIMPs) were reported in idiopathic pulmonary fibrosis (IPF) and in acute respiratory distress syndrome (ARDS) [4-6]. Few studies have focused on AoDILD in collagen diseases. Therefore, we investigated the serum biomarker profile of AoDILD in collagen diseases in order to shed light on pathogenesis and markers informative for disease severity or predicting outcome.

## Methods

### Patients and sera

Twenty-five patients with collagen diseases (mean age ± standard deviation (SD): 65.9 ± 10.8 years; 11 men) were admitted to Sagamihara Hospital between 2001 and 2010, because of AoDILD requiring corticosteroid pulse therapy. AoDILD was defined as acute onset and progression within a month, the presence of clinical symptoms (fever, dry cough, or dyspnea), hypoxia, and computed tomography findings of ILD [3]. Patients with evidence of apparent bacterial infection or heart disease were excluded. These 25 collagen disease patients with AoDILD include 9 AE-ILD, 16 DI-ILD, and no *Pneumocystis* pneumonia. In this study, AoDILD was classified to AE-ILD, DI-ILD, and *Pneumocystis* pneumonia as following: *Pneumocystis* pneumonia was defined by the presence of *P. jirovecii* organisms detected by polymerase chain reaction for *P. jirovecii* or Grocott stain from bronchoalveolar lavage fluids or sputa of patients, DI-ILD was defined as AoDILD with treatment of DI-ILD causing drugs (disease-modifying anti-rheumatic drugs or immunosuppressants excluding corticosteroid) at onset after the exclusion of *Pneumocystis* pneumonia, AE-ILD was defined as AoDILD without treatment of DI-ILD causing drugs at onset, but with underlying CVD-ILD, after the exclusion of *Pneumocystis* pneumonia and DI-ILD. These 16 patients with DI-ILD were treated at onset with methotrexate (n = 10), gold sodium thiomalate (n = 1), tacrolimus (n = 2), cyclophosphamide (n = 1), or etanercept (n = 2). Sera were collected on admission, and in the stable state, at least three months before admission. They were classified according to the American College of Rheumatology criteria for RA [7], systemic sclerosis (SSc) [8], and Bohan’s criteria for polymyositis/dermatomyositis (PM/DM) [9]. Diagnoses of the patients included 20 RA, 2 SSc, and 3 PM/DM. These 25 collagen disease patients with AoDILD include 11 patients who died and 14 who survived, during the course of AoDILD. The major cause of death of these 11 patients would be respiratory failure due to AoDILD, though the complication of infection due to repetitive corticosteroid pulse therapy could not be completely excluded. This study was reviewed and approved by Sagamihara Hospital Research Ethics Committee. Written informed consent was obtained from all study participants except those already deceased before starting this study. The serum samples collected before this study were anonymized in a fashion preventing any link with the patients’ identification and their analysis approved on that condition by Sagamihara Hospital Research Ethics Committee. This study was conducted in accordance with the principles expressed in the Declaration of Helsinki.

### Biomarker immunoassay

RayBio human cytokine antibody array (RayBiotech, Norcross, GA) was used for detection of 274 biomarkers in pooled sera of two states, AoDILD and stable, according to the manufacturer’s protocol. The Bio-Plex suspension array system (Bio-rad, Hercules, CA) was used for detection of biomarkers in individual sera from patients in the two different states, without pooling. Fluorokine MAP multiplex kits (R&D Systems, Minneapolis, MN) were used for detection of TIMP-1, TIMP-2, TIMP-3, and TIMP-4. Milliplex map kits (Millipore, Billerica, MA) were used for detection of transforming growth factor (TGF)-β1, TGF-β2, TGF-β3, leptin, osteopontin, and insulin. Procarta cytokine plex kits (Affymetrix, Santa Clara, CA) were used for detection of MMP-3, MMP-9, epidermal growth factor (EGF), (IL)-17F, IL-1α, IL-1 receptor antagonist (IL-1RA), IL-2, IL-2 receptor α (IL-2Rα), IL-6, Fas, Fas ligand (FasL), tumor necrosis factor (TNF)α, MMP-1, MMP-12, MMP-7, MMP-13, MMP-8, leukaemia inhibitory factor (LIF), and migration inhibitory factor (MIF).

### Statistical analysis

Differences in patient characteristics were analyzed by Mann-Whitney U test or Fisher’s exact test using 2×2 contingency tables. Wilcoxon signed-rank test or Mann-Whitney U test was performed in the comparison of laboratory findings and biomarker assay results. It was defined statistical significance as *P* < 0.05.

## Results

### Characteristics of collagen disease patients with AoDILD

In 84% (n = 21) of the patients with AoDILD, underlying CVD-ILD had been detected prior to the onset of AoDILD. Mortality of patients during the AoDILD state was 44% (n = 11). Lactate dehydrogenase, blood urea nitrogen, KL-6, and surfactant protein-D (SP-D) were more increased in AoDILD than in the stable state (Additional file 1: Table S1). Albumin was decreased in the AoDILD state compared to these patients in the stable state.

### Biomarker immunoassay

**Figure 1:**
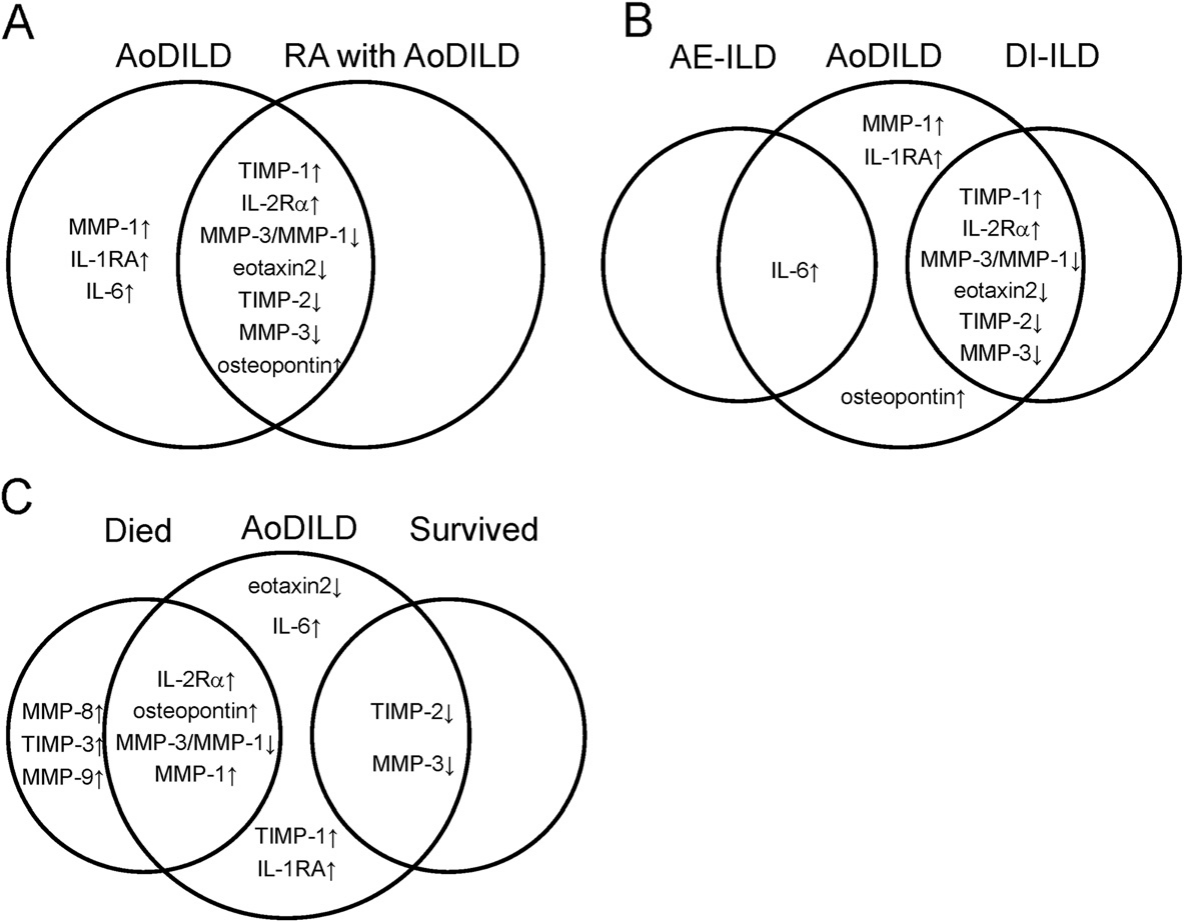
**Biomarker profile of acute-onset diffuse interstitial lung disease (AoDILD).** Increased and decreased biomarkers in AoDILD state of collagen disease and rheumatoid arthritis (RA) patients were indicated in **A**. Increased and decreased biomarkers in AoDILD state of acute exacerbation of ILD (AE-ILD) and drug-induced interstitial lung disease (DI-ILD) patients were indicated in **B**. Increased and decreased biomarkers in AoDILD state of patients who subsequently died and those who survived were indicated in **C**.

Sera were pooled from 25 collagen disease patients in each state, i.e. stable and AoDILD. We assessed the presence of 274 biomarkers in these two pooled sera. Eighteen of them were found to be present at less than 0.67 times the level in the pooled sera at AoDILD compared to the stable state (Additional file 2: Table S2). Five biomarkers were present in AoDILD sera at levels more than 1.5 times the stable state. In addition to these 23 up-regulated or down-regulated biomarkers, several biomarkers were included from the results of previous studies on IPF or ARDS for candidates of further analysis [4,6,10-18]. Thirty one biomarkers were selected based on the availability of bead-based immunoassay for more detailed analysis. Up-regulated soluble TNF receptor II in this pooled assay would be derived from the administered recombinant soluble TNF receptor II-Fc fusion protein, etanercept. Because administration of antibody or recombinant cytokine receptors skews the cytokine profile [19], sera from the 2 RA patients treated with etanercept were excluded from the following individual biomarker analysis. The serum biomarker levels of the 23 collagen disease patients in the stable state and at AoDILD without pooling are shown in Table 1 and Additional file 3: Figure S1. Serum TIMP-1, osteopontin, IL-1RA, IL-2Rα, IL-6, and MMP-1 levels were significantly increased in the AoDILD state (Table 1, Figure 1A), whereas TIMP-2, MMP-3, and eotaxin 2 levels were decreased. The ratio of MMP-3 to MMP-1 was reduced in the AoDILD state in these patients.

**Table 1:** Biomarker levels in individual serum without pooling from collagen disease patients in the stable and AoDILD states

**Biomarker (pg/ml)**	**Stable**		**AoDILD**		***P***
TIMP-1	207843	(57972)	285600	(98476)	0.015
TIMP-2	121481	(22995)	101922	(18568)	0.006
TIMP-3	15662	(11925)	15653	(7974)	0.196
TIMP-4	2908	(941)	2874	(837)	0.906
MMP-3	854165	(668239)	499442	(312919)	0.010
MMP-9	2615399	(1577133)	2837226	(1619793)	0.563
TGF-β1	57391	(12908)	52821	(16415)	0.101
TGF-β2	2475	(889)	2201	(997)	0.101
TGF-β3	0	(0)	0	(0)	1.000
Leptin	6424	(6663)	6275	(8249)	0.670
Osteopontin	14603	(12657)	30216	(33636)	0.012
Insulin	633	(475)	589	(330)	0.961
EGF	131	(124)	130	(189)	0.355
Eotaxin2	4497	(3166)	4223	(5065)	0.018
Eotaxin3	44	(71)	37	(52)	0.625
IL-17 F	125	(340)	168	(519)	0.761
IL-1α	61	(248)	137	(607)	1.000
IL-1RA	156	(330)	401	(490)	0.049
IL-2	41	(121)	77	(246)	0.427
IL-2Rα	3623	(2134)	7469	(6483)	0.001
IL-6	103	(234)	363	(794)	0.035
Fas	2237	(9967)	3450	(13337)	0.715
FasL	68	(63)	74	(74)	0.763
TNFα	841	(1953)	1052	(2676)	0.821
MMP-1	69662	(86194)	75510	(58379)	0.016
MMP-12	4992	(14919)	7406	(23641)	0.500
MMP-7	5874	(5153)	7871	(11084)	0.627
MMP-13	37860	(74534)	52019	(111830)	0.754
MMP-8	25817	(17712)	48700	(54791)	0.144
LIF	57	(150)	121	(353)	0.441
MIF	17352	(34236)	23178	(29813)	0.136
MMP-3/MMP-1	22.04	(18.56)	15.00	(19.61)	0.0208

AoDILD: acute-onset diffuse interstitial lung disease. Average values of each group are shown. Standard deviations are shown in parenthesis. Differences were tested by Wilcoxon signed-rank test.

### Serum biomarker profile of the RA patients with AoDILD

We next explored biomarkers associated with AoDILD in the patients with RA. Characteristics of the RA patients (n = 18) are compared with that of collagen disease patients (n = 23) in Additional file 4: Table S3. The laboratory findings of the RA patients in the stable state and in the AoDILD state are shown in Table 2. KL-6, SP-D, and lactate dehydrogenase were increased in AoDILD of patients with RA. Albumin was decreased in AoDILD of patients with RA.

**Table 2:** Laboratory findings and serum biomarker levels of the RA patients in stable and AoDILD states

	**RA patients (n = 18)**		
	**Stable**	**AoDILD**	***P***
White Blood Cell count(X1000/ml)	10.5 (3.8)	12.1 (3.4)	0.1578
Red Blood Cell count(X10^6^/ml)	4.2 (0.5)	4.1 (0.6)	0.8506
Hemoglobin(g/dl)	12.6 (2.0)	12.3 (1.8)	0.6378
Hematocrit(%)	39.4 (5.5)	37.4 (5.3)	0.4703
Platelet(X1000/ml)	304.1 (121.2)	285.9 (108.4)	0.7064
Albumin(g/dl)	3.9 (0.4)	3.2 (0.6)	0.0052
Aspartate Aminotransferase(IU/l)	32.6 (39.8)	38.6 (27.2)	0.1167
Alanine Aminotransferase(IU/l)	28.0 (36.3)	26.4 (28.8)	0.8068
Lactate Dehydrogenase(IU/l)	224.7 (39.1)	371.8 (143.4)	0.0052
Alkaline Phosphatase(IU/l)	275.4 (129.1)	260.4 (93.3)	0.9721
γ-glutamyltransferase(IU/l)	33.5 (25.4)	110.9 (309.5)	0.1361
Creatinine(mg/dl)	0.6 (0.1)	0.7 (0.2)	0.3627
Blood Urea Nitrogen(mg/dl)	14.2 (2.8)	17.3 (4.6)	0.1005
C-reactive protein(mg/dl)	3.6 (3.6)	11.1 (9.3)	0.1094
KL-6(IU/ml)	447.9 (244.3)	829.2 (473.8)	0.0052
Surfactant Protein-D (SP-D)(U/ml)	74.1 (51.1)	169.3 (148.0)	0.0157
TIMP-1(pg/ml)	196945 (49413)	302152 (102113)	0.0071
TIMP-2(pg/ml)	117585 (23704)	99258 (16673)	0.0231
MMP-3(pg/ml)	910356 (732400)	451701 (312564)	0.0057
Osteopontin(pg/ml)	17095 (13228)	36200 (35682)	0.0198
Eotaxin2(pg/ml)	4482 (3387)	3308 (4017)	0.0108
IL-1RA(pg/ml)	187 (365)	401 (359)	0.0759
IL-2Rα(pg/ml)	3842 (2128)	8624 (6810)	0.0025
IL-6(pg/ml)	123 (260)	383 (852)	0.1119
MMP-1(pg/ml)	81755 (93582)	87509 (60314)	0.0582
MMP-8(pg/ml)	28379 (17114)	55764 (59399)	0.1841
MMP-3/MMP-1	20.66 (20.57)	12.83 (20.91)	0.0249

RA: rheumatoid arthritis, AoDILD: Acute-onset diffuse interstitial lung disease. Average values of each group are shown. Standard deviations are shown in parenthesis. Differences were tested by Wilcoxon signed-rank test.

While the serum TIMP-1, osteopontin, and IL-2Rα levels were increased in patients with RA in the AoDILD state (Table 2, Figure 1A), TIMP-2, MMP-3, and eotaxin 2 were decreased. The ratio of MMP-3 to MMP-1 was also reduced in the AoDILD state in these patients. These biomarker profiles were similar to that of collagen disease patients with AoDILD.

### Serum biomarker profile of the patients with AE-ILD or DI-ILD

We also explored biomarkers associated with AoDILD in the patients with AE-ILD or DI-ILD. Characteristics of the AE-ILD (n = 9) and DI-ILD patients (n = 14) are shown in Additional file 5: Table S4. The laboratory findings of the patients with AE-ILD or DI-ILD in the stable state and in the AoDILD state are shown in Table 3. KL-6 was increased in AoDILD of patients with AE-ILD. Albumin and hematocrit were decreased in AoDILD of patients with AE-ILD. KL-6 and lactate dehydrogenase were increased in AoDILD of patients with DI-ILD.

**Table 3:** Laboratory findings and serum biomarker levels of collagen disease patients with AE-ILD or DI-ILD in stable and AoDILD states

	**AE-ILD (n = 9)**			**DI-ILD (n = 14)**		
	**Stable**	**AoDILD**	***P***	**Stable**	**AoDILD**	***P***
White Blood Cell count (X1000/ml)	10.9 (2.7)	11.8 (2.6)	0.2135	10.3 (4.0)	12.1 (3.8)	0.2604
Red Blood Cell count (X10^6^/ml)	4.2 (0.9)	4.4 (0.7)	0.9528	4.2 (0.5)	4.1 (0.6)	0.5536
Hemoglobin (g/dl)	13.4 (1.2)	12.7 (2.5)	0.2936	12.6 (2.3)	12.2 (1.9)	0.5147
Hematocrit (%)	42.1 (3.1)	38.9 (6.4)	0.0152	38.9 (6.4)	36.9 (5.9)	0.9528
Platelet (X1000/ml)	280.3 (58.0)	299.5 (110.0)	0.3139	318.2 (139.3)	293.5 (118.8)	0.8590
Albumin (g/dl)	4.1 (0.3)	3.6 (0.7)	0.0077	3.9 (0.4)	3.2 (0.6)	0.0687
Aspartate Aminotransferase (IU/l)	41.2 (47.9)	44.4 (32.4)	0.6784	23.2 (4.6)	31.8 (13.6)	0.1282
Alanine Aminotransferase (IU/l)	36.6 (43.2)	39.0 (36.2)	0.4838	19.7 (6.8)	18.0 (6.0)	0.6784
Lactate Dehydrogenase (IU/l)	250.7 (45.3)	311.6 (86.2)	0.1386	215.2 (23.8)	383.6 (158.3)	0.0209
Alkaline Phosphatase (IU/l)	278.1 (141.2)	239.3 (97.9)	0.7794	243.8 (89.5)	261.8 (90.2)	0.9528
γ-glutamyltransferase (IU/l)	52.2 (44.4)	179.5 (392.2)	0.1551	26.3 (18.0)	31.1 (14.9)	0.4990
Creatinine (mg/dl)	0.7 (0.1)	0.7 (0.1)	0.8127	0.6 (0.1)	0.7 (0.2)	0.8127
Blood Urea Nitrogen (mg/dl)	13.9 (2.3)	17.1 (6.2)	0.1097	14.4 (3.1)	16.8 (3.5)	0.4008
C-reactive protein (mg/dl)	2.1 (2.3)	6.4 (10.1)	0.1097	3.9 (4.1)	11.2 (7.7)	0.2135
KL-6 (U/ml)	2078.1 (2525.4)	2105.8 (2495.5)	0.0280	431.0 (256.6)	799.7 (425.9)	0.0208
Surfactant Protein-D (ng/ml)	160.7 (126.5)	194.7 (123.2)	0.1763	76.4 (53.5)	170.5 (163.6)	0.0619
TIMP-1 (pg/ml)	192899 (54474)	245030 (93165)	0.3105	218303 (58054)	313999 (91931)	0.0166
TIMP-2 (pg/ml)	123534 (24219)	100422 (12693)	0.1763	120044 (21985)	102973 (21694)	0.0125
TIMP-3 (pg/ml)	16813 (5411)	18791 (9923)	0.7353	14857 (14822)	13457 (5240)	0.1097
MMP-3 (pg/ml)	587882 (245290)	628244 (328962)	0.5940	1025348 (787427)	416640 (271619)	0.0019
MMP-9 (pg/ml)	2852316 (1698909)	2800269 (1205613)	0.5940	2463095 (1473636)	2860985 (1837002)	0.5509
Osteopontin (pg/ml)	12464 (10678)	28442 (35397)	0.0663	15979 (13604)	31356 (32402)	0.0736
Eotaxin2 (pg/ml)	3953 (2312)	5311 (5724)	0.3139	4846 (3566)	3523 (4453)	0.0258
IL-1RA (pg/ml)	94 (111)	417 (653)	0.0796	196 (409)	390 (346)	0.2209
IL-2Rα (pg/ml)	2611 (1669)	3689 (2047)	0.0506	4274 (2147)	9899 (7159)	0.0076
IL-6 (pg/ml)	37 (47)	238 (422)	0.0209	145 (290)	444 (952)	0.2455
MMP-1 (pg/ml)	26532 (18626)	44551 (43273)	0.1097	97389 (100090)	95411 (58164)	0.0962
MMP-8 (pg/ml)	24640 (18134)	49581 (74715)	0.3743	26573 (17394)	48134 (36641)	0.1401
MMP-3/MMP-1	29.78 (16.71)	29.11 (24.88)	0.7671	17.07 (17.98)	5.92 (4.81)	0.0043

AoDILD: Acute-onset diffuse interstitial lung disease, AE-ILD: acute exacerbation of interstitial lung disease, DI-ILD: drug-induced interstitial lung disease. Average values of each group are shown. Standard deviations are shown in parenthesis. Differences were tested by Wilcoxon signed-rank test.

The serum IL-6 level was significantly increased in patients with AE-ILD in the AoDILD state (Table 3, Figure 1B). While the serum TIMP-1 and IL-2Rα levels were significantly increased in patients with DI-ILD in the AoDILD state (Table 3, Figure 1B), TIMP-2, MMP-3, and eotaxin 2 were decreased. The ratio of MMP-3 to MMP-1 was also reduced in the AoDILD state in these patients. These skewed biomarker profiles suggest differences in the pathogenesis of AoDILD in patients with AE-ILD or DI-ILD.

### Association of serum biomarker profile with outcome

The characteristics of 23 collagen disease patients with AoDILD are shown in Additional file 6: Table S5. There were no significant differences between those who died and those who survived. KL-6 and SP-D were increased and red blood cell count, hemoglobin, and hematocrit were decreased in the AoDILD state of patients who died (n = 9, Table 4). Lactate dehydrogenase, blood urea nitrogen and KL-6 were increased and albumin decreased in the AoDILD state in patients who survived (n = 14). TIMP-3, MMP-9, osteopontin, IL-2Rα, MMP-1, and MMP-8 levels were significantly increased in the AoDILD state in the deceased patients. The ratio of MMP-3 to MMP-1 was decreased in the AoDILD state in the deceased patients (Table 4, Figure 1C). On the other hand, the serum TIMP2 and MMP-3 levels were decreased in the AoDILD state in patients who survived. Thus, biomarker profiles were different in patients with different outcomes.

**Table 4:** Laboratory findings and serum biomarker levels of collagen disease patients with different clinical outcome in stable and AoDILD states

	**Died (n = 9)**			**Survived (n = 14)**		
	**Stable**	**AoDILD**	***P***	**Stable**	**AoDILD**	***P***
White Blood Cell count(X1000/ml)	8.5 (1.7)	10.4 (1.8)	0.138	11.4 (3.6)	13.0 (3.7)	0.279
Red Blood Cell count(X10^6^/ml)	4.3 (0.3)	3.9 (0.7)	0.043	4.1 (0.8)	4.4 (0.6)	0.069
Hemoglobin(g/dl)	13.7 (1.2)	11.7 (2.7)	0.043	12.7 (2.0)	12.9 (1.7)	0.108
Hematocrit(%)	41.5 (3.5)	35.6 (6.7)	0.043	40.1 (5.8)	39.2 (5.4)	0.917
Platelet(X1000/ml)	266.0 (62.5)	310.1 (131.2)	0.281	312.1 (119.0)	287.1 (102.4)	0.972
Albumin(g/dl)	3.9 (0.2)	3.0 (0.6)	0.068	4.1 (0.5)	3.6 (0.5)	0.005
Aspartate Aminotransferase(IU/l)	54.2 (60.6)	43.3 (34.2)	0.893	23.8 (6.8)	33.5 (14.0)	0.068
Alanine Aminotransferase(IU/l)	47.0 (55.2)	34.1 (38.9)	0.500	20.8 (7.8)	22.6 (11.1)	0.480
Lactate Dehydrogenase(IU/l)	234.0 (32.2)	361.8 (92.8)	0.138	232.5 (43.0)	346.2 (158.2)	0.039
Alkaline Phosphatase(IU/l)	320.2 (169.8)	224.9 (67.2)	0.144	238.2 (82.4)	268.5 (103.1)	0.221
γ-glutamyltransferase(IU/l)	42.4 (32.7)	179.1 (413.2)	0.419	38.1 (37.5)	42.8 (44.2)	0.142
Creatinine(mg/dl)	0.7 (0.1)	0.7 (0.2)	0.419	0.7 (0.1)	0.7 (0.1)	0.422
Blood Urea Nitrogen(mg/dl)	13.1 (2.5)	15.1 (5.2)	0.715	14.5 (2.7)	18.1 (4.3)	0.039
C-reactive protein(mg/dl)	0.8 (0.5)	12.0 (8.6)	0.080	3.8 (3.7)	7.3 (9.0)	0.311
KL-6(U/ml)	1364.1 (2423.4)	1565.4 (2498.4)	0.025	837.5 (936.0)	1173.8 (898.0)	0.017
Surfactant Protein-D(ng/ml)	136.8 (125.6)	196.0 (114.4)	0.036	87.1 (60.4)	169.6 (167.8)	0.203
TIMP-1(pg/ml)	231906 (68528)	346576 (72337)	0.080	197816 (49624)	260193 (96764)	0.084
TIMP-2(pg/ml)	118406 (27648)	105669 (20160)	0.345	122762 (20616)	100361 (17629)	0.012
TIMP-3(pg/ml)	10668 (6632)	15759 (12498)	0.043	17743 (12977)	15609 (4998)	0.790
MMP-3(pg/ml)	752633 (495718)	513110 (247738)	0.214	919436 (751496)	490655 (348157)	0.030
MMP-9(pg/ml)	1769108 (1012232)	3358493 (1977141)	0.011	3159443 (1634403)	2502127 (1229009)	0.140
Osteopontin(pg/ml)	13588 (9212)	51660 (44658)	0.008	15256 (14407)	16430 (9537)	0.551
Eotaxin 2(pg/ml)	4854 (4321)	4320 (5943)	0.110	4267 (2079)	4160 (4408)	0.096
IL-1RA(pg/ml)	224 (463)	657 (559)	0.086	112 (190)	236 (352)	0.445
IL-2Rα(pg/ml)	3827 (2213)	8408 (4328)	0.011	3492 (2072)	6865 (7488)	0.064
IL-6(pg/ml)	156 (339)	710 (1157)	0.066	69 (115)	140 (222)	0.328
MMP-1(pg/ml)	35772 (19709)	73909 (39388)	0.011	91449 (103647)	76538 (67816)	0.397
MMP-8 (pg/ml)	14814 (12583)	51014 (40613)	0.008	32889 (16905)	47213 (62177)	0.826
MMP-3/MMP-1	23.67 (18.54)	10.29 (8.39)	0.028	21.00 (18.50)	18.02 (23.73)	0.221

AoDILD: Acute-onset diffuse interstitial lung disease. Average values of each group are shown. Standard deviations are shown in parenthesis. Differences were tested by Wilcoxon signed-rank test.

## Discussion

Many studies have reported on biomarker profiles in IPF or ARDS patients [4-6]. The roles of MMPs and TIMPs in IPF or ARDS have been reported [10-12]. Involvement of other biomarkers in IPF or ARDS, including osteopontin [13], eotaxin 2 (CCL24) [14], IL-1RA [15,16], IL- 2Rα [17], and IL-6 [18], were also inferred. The present study reports novel biomarker profiles of AoDILD occurring in collagen disease patients, though a few of these biomarkers have been analyzed previously in such patients with chronic CVD-ILD [20,21]. Administration of the cytokines found to be decreased, or blocking cytokines found to be increased with antibodies or inhibitors could represent new therapeutic approaches for AoDILD in collagen disease patients [22].

We also propose that serum biomarker patterns could represent prognostic markers for AoDILD in collagen diseases. Several studies reported that the expression of MMP-1 and MMP-3 by fibroblasts and chondrocytes was correlated [12,23]. This was measured as the ratio of MMP-3 to MMP-1 and was maintained in the sera of patients who survived, but not in those who died (Table 4, Figure 1C). Similarly, different biomarker profiles were seen in deceased and surviving patients. These biomarkers were up-regulated in the sera of the patients with AoDILD who died (TIMP-3, MMP-9, osteopontin, IL-2Rα, MMP-1, and MMP-8, Figure 1C) or down-regulated in the sera of the patients with AoDILD who survived (TIMP2 and MMP-3). These biomarker molecules might accelerate the progress of AoDILD and could be targets blocked with antibodies or inhibitors for the treatment. Since there were no oppositely regulated biomarker molecules, we could not find any molecules that might prevent the progress of AoDILD. Role of other molecules in the pathological state of AoDILD, such as TIMP-1, eotaxin 2, IL-1RA, or IL-6 could not be estimated in this study. Thus, the MMP-3 to MMP-1 ratio, and levels of TIMP-3, MMP-9, and osteopontin could be prognostic markers for AoDILD in collagen diseases.

The serum osteopontin (*P* = 0.0233, Mann-Whitney U test), IL-1RA (*P* = 0.0350), and IL-6 (*P* = 0.0320) levels were higher in patients who died in the AoDILD state, compared with those who survived (Table 4). The serum MMP-9 (*P* = 0.0376, Mann-Whitney U test) and MMP-8 (*P* = 0.0167) levels were higher in patients who survived in the stable state, compared with those who died (Table 4). These data suggest that some biomarkers could represent prognosis of the patients with AoDILD, without comparison of the paired serum levels between stable and AoDILD states.

The ratio of MMP-3 to MMP-1 was maintained in the sera of AE-ILD patients (Table 3, Figure 1B). However, the correlation was no longer present in DI-ILD patients (Table 3). In addition, serum profiles of eotaxin 2 and MMP-3 were completely different in patients with AE-ILD and DI-ILD. The serum IL-2Rα, (*P* = 0.0406, Mann-Whitney U test) and MMP-1 (*P* = 0.0140) levels were higher in patients with DI-ILD in the AoDILD state, compared with those with AE-ILD (Table 3). The ratio of MMP-3 to MMP-1 was lower in patients with DI-ILD in the AoDILD state, compared with those with AE-ILD (*P* = 0.0068, Table 3). The serum MMP-1 (*P* = 0.0016, Mann-Whitney U test) level was higher in patients with DI-ILD in the stable state, compared with those with AE-ILD (Table 3). Thus, our findings suggest the possibility of different pathogenesis of AE-ILD and DI-ILD. Because of the limited sample size of this study, the expression pattern of these biomarkers needs to be confirmed in future studies.

## Conclusions

To our knowledge, this is the first report of biomarker profiling in AoDILD occurring in collagen disease patients. Our findings support the role of serum biomarker profiles as prognosis markers for AoDILD.

## Abbreviations

AE-ILD: Acute exacerbation of ILD; AoDILD: Acute-onset diffuse ILD; ARDS: Acute respiratory distress syndrome; CVD-ILD: Collagen vascular disease-associated ILD; DI-ILD: Drug-induced ILD; EGF: Epidermal growth factor; FasL: Fas ligand; IL: Interleukin; IL-1RA: IL-1 receptor antagonist; IL-2Rα: Interleukin-2 receptor α; ILD: Interstitial lung disease; IPF: Idiopathic pulmonary fibrosis; LIF: Leukaemia inhibitory factor; MIF: Migration inhibitory factor; MMP: Matrix metalloproteinase; PM/DM: Polymyositis/dermatomyositis; RA: Rheumatoid arthritis; SD: Standard deviation; SP-D: Surfactant protein-D; SSc: Systemic sclerosis; TGF: Transforming growth factor; TIMP: Tissue inhibitor of metalloproteinase; TNF: Tumor necrosis factor.

## Competing interests

HF has the following conflicts. The following funders are supported in whole or in part by the subsequent pharmaceutical companies. The Japan Research Foundation for Clinical Pharmacology is run by Daiichi Sankyo, the Takeda Science Foundation is supported by an endowment from Takeda Pharmaceutical Company and the Nakatomi Foundation was established by Hisamitsu Pharmaceutical Co., Inc. The Daiwa Securities Health Foundation was established by Daiwa Securities Group Inc. ST was supported by research grants from pharmaceutical companies: Abbott Japan Co., Ltd., Astellas Pharma Inc., AstraZeneca K.K., Bristol-Myers Squibb Co Ltd., Chugai Pharmaceutical Co., Ltd., Eisai Co., Ltd., Medical & Biological Laboratories Co., Ltd, Mitsubishi Tanabe Pharma Corporation, Merck Sharp and Dohme Inc., Pfizer Japan Inc., Takeda Pharmaceutical Company Limited, and Teijin Pharma Limited. The other authors declare no financial or commercial conflict of interest.

## Authors’ contributions

SO and HF carried out immunoassay, participated in the design of the study, performed the statistical analysis, and wrote the manuscript. KS, HF, and ST recruited Japanese patients with collagen diseases and collected clinical information. HH collected serum samples. NF, NT, and ST conceived the study, participated in its design and coordinated and helped to draft the manuscript. All authors read and approved the final manuscript.

## Supplementary Material

Additional file 1: Table S1 Laboratory findings of collagen disease patients in the stable and the AoDILD state. Click here for file

Additional file 2: Table S2 Cytokine expression ratios in sera of the patients between stable and AoDILD states. Click here for file

Additional file 3: Figure S1 Biomarker levels in individual serum without pooling from collagen disease patients in the stable and AoDILD states. Click here for file

Additional file 4: Table S3 Characteristics of collagen disease or RA patients with AoDILD. Click here for file

Additional file 5: Table S4 Characteristics of collagen disease patients with AE-ILD or DI-ILD. Click here for file

Additional file 6: Table S5 Characteristics of collagen disease patients with AoDILD with different clinical outcome. Click here for file
