# Supplementary material for: Serum biomarker analysis of collagen disease patients with acute-onset diffuse interstitial lung disease
Source: BMC Immunol. 2013 Feb 14;14:9. doi: 10.1186/1471-2172-14-9 (PMC3598392; doi:10.1186/1471-2172-14-9)
Supplement: Additional file 1: Table S1 — Laboratory findings of collagen disease patients in the stable and the AoDILD state. [file 1471-2172-14-9-S1.docx]

Supplementary Table 1. Laboratory findings of collagen disease patients in the stable and the AoDILD state.

|  |  | Stable | AoDILD | *P* |
| --- | --- | --- | --- | --- |
| White Blood Cell count | X1000/μl | 10.4 (3.3) | 11.8 (3.3) | 0.079 |
| Red Blood Cell count | X10^6^/μl | 4.1 (0.7) | 4.1 (0.7) | 0.911 |
| Hemoglobin | g/dl | 12.8 (1.9) | 12.2 (2.4) | 0.763 |
| Hematocrit | % | 40.0 (5.4) | 37.3 (6.9) | 0.093 |
| Platelet | X1000/μl | 291.2 (119.2) | 287.3 (120.0) | 0.780 |
| Albumin | g/dl | 4.0 (0.4) | 3.4 (0.6) | 0.002 |
| Aspartate Aminotransferase | IU/l | 32.7 (33.7) | 37.2 (24.4) | 0.356 |
| Alanine Aminotransferase | IU/l | 31.2 (34.4) | 27.0 (25.7) | 0.825 |
| Lactate Dehydrogenase | IU/l | 234.8 (43.1) | 345.9 (137.9) | 0.012 |
| Alkaline Phosphatase | IU/l | 266.7 (115.6) | 257.2 (94.4) | 0.528 |
| γ-glutamyltransferase | IU/l | 39.9 (34.5) | 93.1 (263.7) | 0.306 |
| Creatinine | mg/dl | 0.7 (0.3) | 0.8 (0.5) | 0.332 |
| Blood Urea Nitrogen | mg/dl | 15.3 (6.9) | 18.3 (8.3) | 0.023 |
| C-reactive protein | mg/dl | 2.9 (3.3) | 8.6 (9.0) | 0.055 |
| KL-6 | U/ml | 1032.8 (1739.4) | 1275.0 (1692.8) | 0.001 |
| Surfactant Protein-D (SP-D) | ng/ml | 105.3 (97.0) | 181.6 (145.6) | 0.011 |

AoDILD: acute-onset diffuse interstitial lung disease. Average values of each group are shown. Standard deviations are shown in parenthesis. Differences were tested by Wilcoxon signed-rank test.
