# Supplementary material for: Serum biomarker analysis of collagen disease patients with acute-onset diffuse interstitial lung disease
Source: BMC Immunol. 2013 Feb 14;14:9. doi: 10.1186/1471-2172-14-9 (PMC3598392; doi:10.1186/1471-2172-14-9)
Supplement: Additional file 2: Table S2 — Cytokine expression ratios in sera of the patients between stable and AoDILD states. [file 1471-2172-14-9-S2.docx]

Supplementary Table2. Cytokine expression ratios in sera of the patients between stable and AoDILD states.

| Cytokine name | Expression ratio |
| --- | --- |
| CK b 8-1 | 0.362963 |
| Leptin | 0.491134 |
| SDF-1 | 0.51188 |
| IGFBP-4 | 0.519346 |
| FGF-6 | 0.519726 |
| IL-2 | 0.532313 |
| MIP-1 delta | 0.540668 |
| MSP-alpha | 0.557882 |
| MCP-4 | 0.580576 |
| MCP-2 | 0.585235 |
| FGF-7 | 0.587529 |
| TGF-beta 3 | 0.610791 |
| IL-2 R alpha | 0.611974 |
| EGF | 0.616713 |
| Eotaxin-2 | 0.623381 |
| Eotaxin-3 | 0.64566 |
| Oncostatin M | 0.65585 |
| IGF-I | 0.665969 |
| VE-Cadherin | 0.677179 |
| IL-17F | 0.677997 |
| GCP-2 | 0.678595 |
| IGFBP-2 | 0.681335 |
| I-309 | 0.685662 |
| IL-11 | 0.688597 |
| IL-1ra | 0.690435 |
| IL-3 | 0.691939 |
| TECK | 0.700868 |
| TGF-alpha | 0.713669 |
| BCMA | 0.71368 |
| MMP-3 | 0.713963 |
| IL-15 | 0.716607 |
| bFGF | 0.729045 |
| IL-4 | 0.730451 |
| LIGHT | 0.733189 |
| IL-12 p70 | 0.734158 |
| GITR-Ligand | 0.740042 |
| VEGF-D | 0.745438 |
| TRAIL R4 | 0.751778 |
| TIM-1 | 0.752461 |
| IGFBP-6 | 0.752843 |
| IL-31 | 0.755356 |
| IL-10 | 0.757661 |
| MMP-1 | 0.759391 |
| SCF | 0.760755 |
| GITR | 0.776373 |
| PDGF-BB | 0.778012 |
| HCC-4 | 0.780594 |
| IL-16 | 0.782735 |
| IL-17 | 0.782888 |
| CRP | 0.783811 |
| IL-5 | 0.787068 |
| IL-13 | 0.790333 |
| IL-8 | 0.796274 |
| MIP-3 beta | 0.798693 |
| BLC | 0.800994 |
| IL-1 beta | 0.804134 |
| Lymphotactin | 0.804251 |
| GCSF | 0.807018 |
| CXCL-16 | 0.807699 |
| IL-7 | 0.810325 |
| IFN-gamma | 0.811433 |
| EGF-R | 0.817036 |
| PIGF | 0.819283 |
| RANTES | 0.82033 |
| TIMP-2 | 0.823362 |
| PARC | 0.823723 |
| BTC | 0.825717 |
| 4-1BB | 0.830443 |
| IL-12 p40 | 0.830457 |
| TNF-alpha | 0.831962 |
| M-CSF | 0.83287 |
| TIMP-4 | 0.835972 |
| NGF R | 0.839427 |
| IL-2 R beta | 0.840677 |
| b-NGF | 0.841917 |
| IL-13 R alpha2 | 0.842351 |
| TACE | 0.842749 |
| BDNF | 0.844308 |
| ALCAM | 0.845336 |
| sgp130 | 0.84647 |
| FSH | 0.8517 |
| Eotaxin | 0.852673 |
| Angiopoietin-2 | 0.856376 |
| IL-6 R | 0.858329 |
| LIMPII | 0.858586 |
| Siglec-9 | 0.86429 |
| IL-5 R alpha | 0.86578 |
| BMP-6 | 0.866052 |
| NrCAM | 0.867139 |
| MPIF-1 | 0.867269 |
| BMP-4 | 0.867337 |
| Acrp30 | 0.869662 |
| NT-3 | 0.873452 |
| TNF-beta | 0.875084 |
| PSA-free | 0.875454 |
| MMP-13 | 0.875951 |
| MIP-1 alpha | 0.876846 |
| SCF R | 0.877499 |
| IL-2 R gamma | 0.880307 |
| M-CSF R | 0.880517 |
| Marapsin | 0.886802 |
| Axl | 0.888907 |
| Cripto-1 | 0.895421 |
| CCL-28 | 0.897926 |
| Thrombopoietin | 0.899307 |
| MCP-3 | 0.899867 |
| MIP-3 alpha | 0.902677 |
| GRO-alpha | 0.903739 |
| DR6(TNFRSF21) | 0.909572 |
| IGF-I SR | 0.910537 |
| Angiogenin | 0.911227 |
| IL-18 R beta | 0.918267 |
| IL-1 RI | 0.918729 |
| RANK | 0.919545 |
| NT-4 | 0.923767 |
| HGF | 0.923791 |
| VCAM-1 | 0.926989 |
| SAA | 0.927007 |
| Galectin-7 | 0.929127 |
| ENA-78 | 0.930343 |
| TGF-beta 1 | 0.93418 |
| IL-28A | 0.938933 |
| VEGF R2 | 0.940244 |
| sTNT RI | 0.943247 |
| PDGF R alpha | 0.943583 |
| CA125 | 0.944585 |
| PSA-total | 0.946306 |
| TGF-beta 2 | 0.948513 |
| MMP-10 | 0.951546 |
| TSLP | 0.952072 |
| Amphiregulin | 0.953712 |
| AgRP | 0.953874 |
| Cathepsin S | 0.95582 |
| IL-9 | 0.955938 |
| PDGF-AB | 0.955976 |
| TRAIL R3 | 0.95814 |
| NCAM-1 | 0.958574 |
| Angiopoietin-1 | 0.959009 |
| SDF-1 beta | 0.966481 |
| PDGF R beta | 0.973196 |
| FGF-9 | 0.983267 |
| MIP-1 beta | 0.983606 |
| Fas/TNFRSF6 | 0.983917 |
| S-100b | 0.984673 |
| Angiostatin | 0.986136 |
| CNTF | 0.99195 |
| IL-1 R4/ST2 | 0.993249 |
| IL-10 R alpha | 0.993793 |
| Fractalkine | 1.000913 |
| FGF-4 | 1.00434 |
| I-TAC | 1.005243 |
| ICAM-2 | 1.007032 |
| CCL14a | 1.007054 |
| Dtk | 1.007877 |
| Osteoprotegerin | 1.010259 |
| BMP-5 | 1.014279 |
| beta IG-H3 | 1.016758 |
| MICA | 1.019104 |
| IL-18 BP alpha | 1.019259 |
| PDGF-AA | 1.020475 |
| IGFBP-3 | 1.020624 |
| Platelet factor 4 | 1.021895 |
| GRO | 1.021904 |
| MMP-2 | 1.023701 |
| CD40 Ligand | 1.024903 |
| Flt-3 Ligand | 1.025146 |
| TARC | 1.025594 |
| DKK-4 | 1.025661 |
| CEACAM-1 | 1.025915 |
| NRG1-beta1 | 1.027122 |
| VEGF | 1.02722 |
| DPPIV | 1.027721 |
| Furin | 1.034549 |
| Trappin-2 | 1.038845 |
| GrowthHormon | 1.039621 |
| Erythropoietin R | 1.042258 |
| LAP | 1.042695 |
| PAI-I | 1.042859 |
| CA15-3 | 1.045873 |
| IL-17R | 1.04666 |
| EG-VEGF | 1.047167 |
| XEDAR | 1.049452 |
| RAGE | 1.051042 |
| ErbB2 | 1.051559 |
| CTACK | 1.05217 |
| GDF-15 | 1.053736 |
| u PAR | 1.053836 |
| GDNF | 1.05444 |
| IL-17C | 1.055298 |
| IL-13 R alpha1 | 1.057571 |
| Adiposin | 1.057631 |
| CD14 | 1.058449 |
| Bate2 M | 1.058536 |
| EDA-A2 | 1.05928 |
| VEGF R3 | 1.065421 |
| MIG | 1.067276 |
| IL29 | 1.067439 |
| Follistatin | 1.06883 |
| MDC | 1.070929 |
| L-Selectin | 1.071186 |
| EpCAM | 1.07913 |
| NAP-2 | 1.087657 |
| Nidogen-1 | 1.088721 |
| Cardiotrophin-1 | 1.090941 |
| VEGF-C | 1.091258 |
| IL-22 | 1.091884 |
| MMP-9 | 1.092164 |
| Decorin | 1.093676 |
| TSH | 1.096454 |
| Activin A | 1.097435 |
| MMP-7 | 1.097827 |
| hCGa, intact | 1.101012 |
| Resistin | 1.103229 |
| PECAM-1 | 1.108337 |
| Thyroglobulin | 1.11381 |
| Luteinizinghormone | 1.114654 |
| CD30 | 1.118353 |
| IP-10 | 1.11928 |
| LYVE-1 | 1.129076 |
| Procalcitonin | 1.129923 |
| E-Selectin | 1.131897 |
| BMP-7 | 1.132681 |
| MCP-1 | 1.139129 |
| Prolactin | 1.141411 |
| Fcr RIIB/C | 1.144673 |
| HB-EGF | 1.146543 |
| GM-CSF | 1.147122 |
| CEA | 1.159739 |
| Tie-1 | 1.166306 |
| ANGPTL4 | 1.168104 |
| Alpha-fetoprotein | 1.169717 |
| Endoglin | 1.172202 |
| Tie-2 | 1.172423 |
| CCL21 | 1.174715 |
| TREM-1 | 1.186155 |
| Siglec-5 | 1.19044 |
| FLRG | 1.190549 |
| Carbonic Anhydrase IX | 1.19461 |
| DKK-1 | 1.19523 |
| Fas Ligand | 1.197916 |
| IL-17B | 1.206554 |
| Osteopontin | 1.206914 |
| B7-1(CD80) | 1.225602 |
| Insulin | 1.228866 |
| Ferritin | 1.233943 |
| HVEM | 1.237641 |
| Shh N | 1.237697 |
| IGF-II | 1.239145 |
| CA19-9 | 1.239215 |
| BCAM | 1.243026 |
| ErbB3 | 1.24385 |
| MICB | 1.262449 |
| DKK-3 | 1.278805 |
| IL-1 alpha | 1.282178 |
| Ubiquitin+1 | 1.285382 |
| ICAM-1 | 1.295521 |
| E-Cadherin | 1.297297 |
| IL-10 R beta | 1.30066 |
| LIF | 1.306936 |
| ICAM-3 | 1.308423 |
| DAN | 1.311048 |
| ACE-2 | 1.328675 |
| CD23 | 1.339686 |
| IL-21 R | 1.36007 |
| IL-1 RII | 1.371901 |
| IL-6 | 1.415094 |
| IGFBP-1 | 1.443675 |
| TRAIL R2 | 1.443725 |
| MMP-8 | 1.478453 |
| CD40 | 1.482666 |
| TIMP-1 | 1.656491 |
| Leptin R | 1.968837 |
| MIF | 2.099618 |
| sTNF RII | 13.084755 |
