# Supplementary material for: Serum biomarker analysis of collagen disease patients with acute-onset diffuse interstitial lung disease
Source: BMC Immunol. 2013 Feb 14;14:9. doi: 10.1186/1471-2172-14-9 (PMC3598392; doi:10.1186/1471-2172-14-9)
Supplement: Additional file 3: Figure S1 — Biomarker levels in individual serum without pooling from collagen disease patients in the stable and AoDILD states. [file 1471-2172-14-9-S3.pdf]

Supplementary Table 3 Characteristics of collagen disease or RA patients with AoDILD.

		AoDILD patients with collagen disease	AoDILD patients with RA
Number		23	18
Male number	n (%)	9 (39.1)	5 (27.8)
Age	year (SD)	65.8 (11.1)	68.5 (10.0)
Underliying CVD-ILD positive	n (%)	20 (87.0)	15 (83.3)
Outcome dead	n (%)	9 (39.1)	7 (38.9)
Corticosteroid administration as prednisolone	mg (SD)	10.2 (11.2)	8.1 (8.7)
Diabetes mellitus complication	n (%)	6 (26.1)	4 (22.2)

AoDILD: acute-onset diffuse interstitial lung disease, RA: rheumatoid arthritis, CVD-ILD: collagen vascular disease-associated interstitial lung disease, SD: standard deviation.
