# Supplementary material for: Serum biomarker analysis of collagen disease patients with acute-onset diffuse interstitial lung disease
Source: BMC Immunol. 2013 Feb 14;14:9. doi: 10.1186/1471-2172-14-9 (PMC3598392; doi:10.1186/1471-2172-14-9)
Supplement: Additional file 4: Table S3 — Characteristics of collagen disease or RA patients with AoDILD. [file 1471-2172-14-9-S4.docx]

Supplementary Table 3 Characteristics of collagen disease or RA patients with AoDILD.

|  |  | AoDILD patients with collagen disease | AoDILD patients with RA |
| --- | --- | --- | --- |
| Number |  | 23 | 18 |
| Male number | n (%) | 9 (39.1) | 5 (27.8) |
| Age | year (SD) | 65.8 (11.1) | 68.5 (10.0) |
| Underliying CVD-ILD positive | n (%) | 20 (87.0) | 15 (83.3) |
| Outcome dead | n (%) | 9 (39.1) | 7 (38.9) |
| Corticosteroid administration as prednisolone | mg (SD) | 10.2 (11.2) | 8.1 (8.7) |
| Diabetes mellitus complication | n (%) | 6 (26.1) | 4 (22.2) |

AoDILD: acute-onset diffuse interstitial lung disease, RA: rheumatoid arthritis, CVD-ILD: collagen vascular disease-associated interstitial lung disease, SD: standard deviation.
