# Supplementary material for: Serum biomarker analysis of collagen disease patients with acute-onset diffuse interstitial lung disease
Source: BMC Immunol. 2013 Feb 14;14:9. doi: 10.1186/1471-2172-14-9 (PMC3598392; doi:10.1186/1471-2172-14-9)
Supplement: Additional file 5: Table S4 — Characteristics of collagen disease patients with AE-ILD or DI-ILD. [file 1471-2172-14-9-S5.docx]

Supplementary Table 4 Characteristics of collagen disease patients with AE-ILD or DI-ILD.

|  |  | AE-ILD | DI-ILD |  |
| --- | --- | --- | --- | --- |
| Number |  | 9 | 14 |  |
| Male number | n (%) | 4 (44) | 5 (36) | 1.0000 |
| Age | year (SD) | 63.4 (10.2) | 67.3 (11.5) | 0.2975* |
| Underliying CVD-ILD positive | n (%) | 9 (100) | 11 (79) | 0.2530 |
| Outcome dead | n (%) | 3 (33) | 6 (43) | 0.6913 |
| Corticosteroid administration as prednisolone | mg (SD) | 15.6 (14.4) | 6.8 (6.7) | 0.0619* |
| Diabetes mellitus complication | n (%) | 3 (33) | 3 (21) | 0.6430 |

AE-ILD: acute exacerbation of interstitial lung disease, DI-ILD: drug-induced interstitial lung disease, SD: standard deviation

Difference was tested by Mann-Whitney's U test or Fisher's exact test using 2X2 contingency tables. *Mann-Whitney's U test was employed.
