# Supplementary material for: Serum biomarker analysis of collagen disease patients with acute-onset diffuse interstitial lung disease
Source: BMC Immunol. 2013 Feb 14;14:9. doi: 10.1186/1471-2172-14-9 (PMC3598392; doi:10.1186/1471-2172-14-9)
Supplement: Additional file 6: Table S5 — Characteristics of collagen disease patients with AoDILD with different clinical outcome. [file 1471-2172-14-9-S6.docx]

Supplementary Table 5. Characteristics of collagen disease patients with AoDILD with different clinical outcome.

| Outcome |  | Died |  | Survived |  | *P* |
| --- | --- | --- | --- | --- | --- | --- |
| Number |  | 9 |  | 14 |  |  |
| Male number | n (%) | 5 | (55.6) | 4 | (28.6) | 0.3826 |
| Age | year (SD) | 66.8 | (10.4) | 65.1 | (11.5) | 0.5699* |
| Underlying CVD-ILD positive | n (%) | 9 | (100.0) | 11 | (78.6) | 0.2530 |
| Corticosteroid administration as prednisolone | mg (SD) | 8.3 | (7.2) | 12.1 | (14.2) | 0.8987* |
| Diabetes mellitus complication | n (%) | 3 | (33.3) | 3 | (21.4) | 0.6430 |

AoDILD: acute-onset diffuse interstitial lung disease, CVD-ILD: collagen vascular disease-associated interstitial lung disease, SD: standard deviation. Difference was tested by Mann-Whitney's U test or Fisher's exact test using 2X2 contingency tables. *Mann-Whitney's U test was employed.
